# A 24 h Age Difference Causes Twice as Much Gene Expression Divergence as 100 Generations of Adaptation to a Novel Environment

**DOI:** 10.3390/genes10020089

**Published:** 2019-01-28

**Authors:** Sheng-Kai Hsu, Ana Marija Jakšić, Viola Nolte, Neda Barghi, François Mallard, Kathrin A. Otte, Christian Schlötterer

**Affiliations:** 1 Institut für Populationsgenetik, Vetmeduni Vienna, 1210 Vienna, Austria; Sheng-Kai.Hsu@vetmeduni.ac.at (S.-K.H.); amjaksic@gmail.com (A.M.J.); viola.nolte@vetmeduni.ac.at (V.N.); barghi.neda@gmail.com (N.B.); mallard@biologie.ens.fr (F.M.); kathrin.anna.otte@gmail.com (K.A.O.); 2 Vienna Graduate School of Population Genetics, Vetmeduni Vienna, 1210 Vienna, Austria

**Keywords:** aging, gene expression evolution, RNA-Seq, *Drosophila*, temperature adaptation

## Abstract

Gene expression profiling is one of the most reliable high-throughput phenotyping methods, allowing researchers to quantify the transcript abundance of expressed genes. Because many biotic and abiotic factors influence gene expression, it is recommended to control them as tightly as possible. Here, we show that a 24 h age difference of *Drosophila simulans* females that were subjected to RNA sequencing (RNA-Seq) five and six days after eclosure resulted in more than 2000 differentially expressed genes. This is twice the number of genes that changed expression during 100 generations of evolution in a novel hot laboratory environment. Importantly, most of the genes differing in expression due to age introduce false positives or negatives if an adaptive gene expression analysis is not controlled for age. Our results indicate that tightly controlled experimental conditions, including precise developmental staging, are needed for reliable gene expression analyses, in particular in an evolutionary framework.

## 1. Introduction

Microarray analysis and, more recently, RNA sequencing (RNA-Seq) have become highly reliable methods for high-throughput gene expression analysis of the whole transcriptome. With a single experiment providing a quantitative readout of the expression of all genes, gene expression analysis is the most cost-effective high-throughput phenotyping method.

The high accuracy and sensitivity of the current methods for transcript quantification provide the unique opportunity to study gene expression differences in a wide range of organisms, across the different stages of a developing organism [1], in different environments [2,3,4,5,6,7,8], or even among individual cells [9]. The analysis of gene expression patterns in contrasting environments is a highly promising approach to study phenotypic plasticity on a high-throughput scale [4,5,6,7,8,10]. While such studies typically focus on a single or a few genotypes, another very popular line of research is the comparison of gene expression patterns among individuals with different genotypes or among different species. Such analyses have been used, for example, for the identification of genetic architectures underlying gene expression regulation [11,12]. Moreover, even the evolutionary forces operating on gene expression patterns have been studied by comparing populations collected from distant habitats [13,14,15,16] or contrasting evolved and ancestral populations [17,18,19]. 

Similar to the impact of environmental and genetic variation on gene expression, dynamic changes during development also provide exciting research questions (e.g., expression profiles of the life cycle [20]). Given the well-documented influence of these factors, it is apparent that they need to be tightly controlled for powerful and unbiased gene expression analyses. Because gene expression analyses of natural populations potentially confound genetic variation, environmental heterogeneity, developmental stage, and possibly even tissue heterogeneities [21], it is advised to measure gene expression in settings that control most, if not all, of these factors. Hence, organisms that can be studied in the laboratory provide the potential for gene expression analyses that are much better controlled and thus more informative, in particular for small expression differences. It has become common practice to control for environmental heterogeneity by maintenance under well-defined conditions for multiple generations to minimize transgenerational effects. More challenging is the control of developmental stage, as genetic variation can also result in changes in developmental rates [22,23]. Hence, the analysis of different genotypes during developmental stages with rapid transcriptomic turnover is extremely arduous. An alternative approach to account for this problem focusses on developmental stages which are characterized by low turnover in gene expression levels.

An early landmark study of gene expression in *Drosophila* indicated that adult flies differ dramatically in gene expression between sexes, but, within a given sex, gene expression is rather stable across different age classes [24]. As a consequence, many gene expression analyses in *Drosophila* combine flies differing in age by up to 15 days in a single sample, for example, [11,18,25,26,27,28]. Since several subsequent studies of senescence in *Drosophila* demonstrated considerable gene expression dynamics during aging [29,30,31,32,33], we were interested in scrutinizing the differences in gene expression between flies differing in age by as little as 24 h.

To provide a scale for gene expression differences which goes beyond statistical significance, we contrasted flies differing 24 h in age to adaptive expression changes after more than 100 generations in a novel temperature regime. Surprisingly, our analysis demonstrated that female *Drosophila simulans* flies differing 24 h in age showed about twice as many differentially expressed genes than females evolved for more than 100 generations. Our results highlight that reliable expression analysis in *Drosophila* adults requires accurate timing of the developmental stage to uncover biologically relevant expression changes. 

## 2. Materials and Methods

### 2.1. RNA-Seq Common Garden Experiment

**Figure 1:**
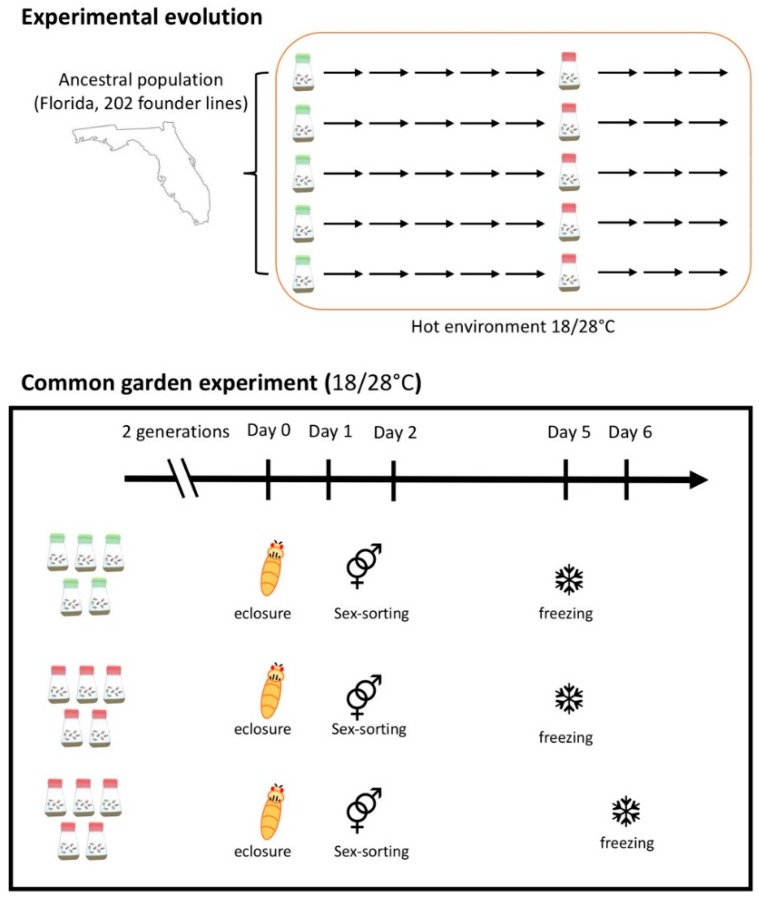
Experimental design. Two hundred and two isofemale lines from a natural *Drosophila simulans* population were used to generate the ancestral population. Five replicates were kept at a population size of 1250 adults with non-overlapping generations in a hot laboratory environment fluctuating between 18 and 28 °C in 12 h dark–12 h light photoperiods. We measured gene expression in a total of 15 samples from 5 reconstituted ancestral populations and 2 copies of each replicate of the evolved population (F103) in a common garden experiment. The two copies of evolved replicates were frozen 24 h apart.

We measured gene expression in two replicated *D. simulans* populations, one ancestral population and the other population adapting for more than 100 generations to a novel hot laboratory environment. Two copies of the evolved populations were frozen on two consecutive days in a common garden experiment (Figure 1). The ancestral population was reconstituted [34] from the same isofemale lines that were collected in Florida, USA, in 2010 and used to seed the experimental evolution study [35]. The common garden experiment was set up after five replicates of the evolved population had adapted for 103 generations to a novel hot laboratory environment fluctuating between 18 and 28 °C in 12 h dark–12 h light photoperiods at a census population size of 1250 adult individuals per population. Five replicates of the reconstituted ancestral population [34] and 10 replicates (two copies of each independently evolved replicate) of the evolved population were reared with controlled egg density (400 eggs/bottle) at the same temperature regimes as during the experimental evolution (28–18 °C in 12 h day–12 h night cycling environment). After two generations in a common environment, during which most transgenerational effects were homogenized between the two populations, 50 females from each replicate of ancestral and evolved populations were collected one day after eclosure. The females were kept at a density of 50 individuals per vial and were checked for male contamination. At day 5 after eclosure, the collected females from each of the five replicates of evolved and ancestral populations were frozen. At day 6 after eclosure, we froze another sample for each replicate of the evolved population, which was exactly 24 h older than the first one. All samples were snap frozen in liquid nitrogen at around 2 p.m. (6 h after the light turned on) and stored at −80 °C until mRNA extraction. Total RNA was extracted from the whole bodies of the flies using a Qiagen RNeasy Universal Plus Mini kit (Qiagen, Hilden, Germany). Libraries were prepared on a Neoprep Library Prep System (Illumina, San Diego, CA, USA) starting from 100 ng total RNA and following the manufacturer’s recommended protocol. Neoprep runs were performed using software version 1.1.0.8 and protocol version 1.1.7.6 with default settings for 15 PCR cycles and an insert size of 200 bp. All libraries for this experiment were prepared in a randomized order on library cards with the same lot number to avoid batch effects. They were sequenced with a 50 bp single-end read protocol on an Illumina HiSeq 2500.

### 2.2. RNA-Seq Data Processing and Analysis

All sequencing reads were trimmed with ReadTools (Version: 1.5.2) [36] based on a quality score of 20 and mapped with GSNAP (Version: 2018-03-25; Parameters: -k 15 -N 1 -m 0.08) [37] to *D. simulans* reference genome [38]. Exon-aligned reads were counted with Rsubread (Version: 1.30.9) [39] based on the annotation [38], and the expression level of each gene was quantified by normalizing total exon-aligned read counts using the TMM method implemented in edgeR (Version: 3.22.5) [40]. Only genes with more than one count per million base pairs in each sample were retained for the analysis to avoid biased analyses. 

We modeled the effect of age and evolution on gene expression variation as: Y=Pop+ε, where Y is the normalized expression value, Pop indicates the combined effect of evolution and age difference with three levels (5-day-old ancestral, 5-day-old evolved, and 6-day-old evolved samples), and ε is the random error. Principal component analysis (PCA) and principal variance component analysis (PVCA) were performed to decompose the variance explained by each factor [41]. Likelihood ratio tests implemented in edgeR were used to perform differential expression analysis on three contrasts: (1) *Age*: 6-day-old evolved samples versus 5-day-old evolved samples, (2) *Evolution*: 5-day-old evolved samples versus 5-day-old ancestral samples, and (3) *Mismatched*: 6-day-old evolved samples versus 5-day-old ancestral samples. Benjamini–Hochberg’s FDR correction was applied to account for multiple testing [42]. Significant genes were required to have both statistical significance and more than 1.25-fold expression difference. Consistent results were obtained using DESeq2 [43] instead of edgeR (Figure S1). For visualization, the expression values of the genes of interest were normalized ($\frac{y-\bar{y}}{\sigma_{y}}$) across samples by each gene.

### 2.3. GO Enrichment Analysis

We performed gene ontology (GO) enrichment analysis of the genes of interest using the topGO package [44] and the database of biological processes for *Drosophila melanogaster*. We applied the default weight01 algorithm which takes GO hierarchy into account for the analysis. Since this method already accounts for multiple testing [45], we directly used the *p*-values provided by topGO. 

### 2.4. Comparison to Gene Expression Changes Associated with Aging

We made use of a recent microarray study which contrasts gene expression patterns of 6-day-old flies to flies which are 13–85 days old in a large cohort of *D. melanogaster* [33]. Like our study, the authors also measured gene expression in a genetically heterogeneous population, rather than in inbred individuals. In this study, 1581 genes were differentially expressed at more than one time point, but only 966 of them had a gene ID corresponding to the expressed genes in our study. We re-performed GO enrichment analysis of these genes using the topGO package to match our study. Fisher’s exact test was applied to test if the genes or the GO terms identified were over-represented in our study.

### 2.5. Availibility of Data and Materials

Sequence reads from this study are available at the European Sequence Read Archive (http://www.ebi.ac.uk/ena/) under the study accession number PRJEB30547. Additionally, we provide our gene expression count table and the script used to perform all the analysis and visualization on GitHub (https://github.com/ShengKaiHsu/D.sim_aging_project).

## 3. Results

Our study contrasted *D. simulans* populations that were genetically similar but differed 24 h in age (contrast: *age*), separated by more than 100 generations of evolution in a novel temperature regime but of similar age (contrast: *evolution*), or diverging in age and history (contrast: *mismatched*). Fifteen RNA-Seq libraries with a total of 240 million short reads (single end, 50 bp) were aligned to the *D. simulans* reference genome [38]. On average, 98.8% of these reads were mapped to the reference genome, and 83.6% were uniquely assigned to annotated exonic regions [38] (Table S1), resulting in a total of 8856 expressed genes.

### 3.1. Age Difference Explains more Variance than Evolutionary Responses

**Figure 2:**
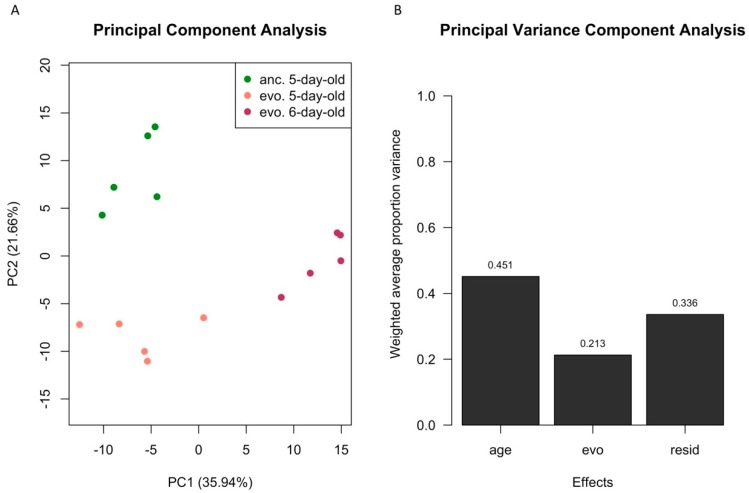
A 24 h age difference explains about twice as much of the variance in gene expression than 100 generations of evolution. (**A**) Multi-dimensional scaling plot with the first and second principal components (PC) shown. (**B**) Weighted average proportion of variance explained by the effect of age and evolution based on principal variance component analysis (PVCA).

In a PCA, the first two PCs explained nearly 60% of the overall variance in gene expression. PC1 accounted for 36% of total variance and separated evolved samples with different ages. PC2 distinguished the evolved samples from their ancestors but explained only 22% of the total variance, which was about half of PC1 (Figure 2A). For a more direct analysis of the principle variables in the experiment, we performed a PVCA [41] and obtained very similar results, with an even larger proportion of the variation being explained by age (Figure 2B). The contrasting variance explained by the two factors was also reflected in the number and magnitude of expression changes. Comparing the absolute expression changes caused by 24 h difference in age and 100 generations of evolution across all expressed genes, we found that age difference contributed to significantly larger gene expression changes than evolution (Wilcoxon’s rank sum test, *p* < 2.2 × 10^−16^, Figure S2). For a more detailed analysis of the underlying genes, we performed a differential gene expression analysis and identified 2051 genes with significant differences in gene expression between populations differing in age by 24 h (FDR < 0.05 and FC > 1.25, contrast: *age*). Only 934 genes changed their expression significantly between the ancestral and evolved populations of the same age (FDR < 0.05 and FC > 1.25, contrast: *evolution*). Therefore, we concluded that as little as 24 h difference in age causes more divergence in gene expression than 100 generations of evolution.

### 3.2. Functional Implications of a 24 h Age Difference or Temperature Adaptation

Out of 2051 age-related genes, 1332 increased in expression, while 719 were downregulated. GO enrichment analysis revealed that genes with increased expression 6 days after eclosure were involved in multiple developmental or pattern formation processes (Table S2), including gonad development (GO:0008406), neuroblast development (GO:0014019), and antennal development (GO:0007469). Furthermore, 9 out of 11 transcription factors (TFs) regulating leg disc proximal/distal pattern formation (GO:0007479) as well as 5 out of 6 TFs involved in wing disc proximal/distal pattern formation (GO:0007473) were upregulated. Because these TFs may be the upstream regulators activating many developmental genes, we suspect their involvement in the secondary development of adult flies. For example, during the first week after eclosure, the number of mushroom body fibers increase in adult fly brains [46], which could be reflected in the GO terms of neuroblast development (GO:0014019), neuroblast fate determination (GO:0007400), neuroblast fate commitment (GO:0014017), mushroom body development (GO:0016319), and brain development (GO:0007420). Processes enriched among downregulated genes affect the cell cycle (e.g., DNA replication, mitosis, meiosis) as well as DNA repair (Table S2), and many of them have been reported to be downregulated during aging [47,48,49]. Despite the fact that the 24 h age difference in our experiment represents only a very small fraction of the typical life of a fly, it is remarkable that many of the observed expression changes were consistent, for both genes and GO enrichment categories, with signatures described for flies aging over substantially larger time spans in a large *D. melanogaster* cohort (Table 1) [33]. 

Among the 934 genes with adaptive expression changes, only 17% (156) increased expression, while the remaining 83% (778) adaptive genes were downregulated. The processes enriched among the upregulated genes included chorion-containing eggshell formation (GO:0007304), eggshell chorion assembly (GO:0007306), and vitelline membrane formation involved in chorion-containing eggshell formation (GO:0007305) (Table S2). These changes probably reflect the documented fecundity increase in the evolved replicates [35]. In addition, several RNA metabolic processes such as nuclear mRNA surveillance (GO:0071028) and nuclear-transcribed mRNA catabolic process (GO:0034427) were enriched among the genes with upregulated expression. This may reflect the importance of mRNA quality control in eukaryotic cells in stressful conditions [50,51]. The 778 genes downregulated during adaptation were mainly involved in cellular signaling processes such as signal transduction (GO:0007165) and regulation of membrane potential (GO:0042391). The GO terms nervous system process (GO:0050877) and mushroom body development (GO:0016319) also point to adaptation processes related to brain functions. In addition to nervous system, enrichment for several other developmental processes including Malpighian tubule development (GO:0072002) and regulation of striated muscle tissue development (GO:0016202) was also decreased during temperature adaptation. Our results suggest that a general delay in multiple developmental processes seems to be beneficial at high temperatures.

### 3.3. Age Difference Confounds Signals of Adaptive Responses

**Figure 3:**
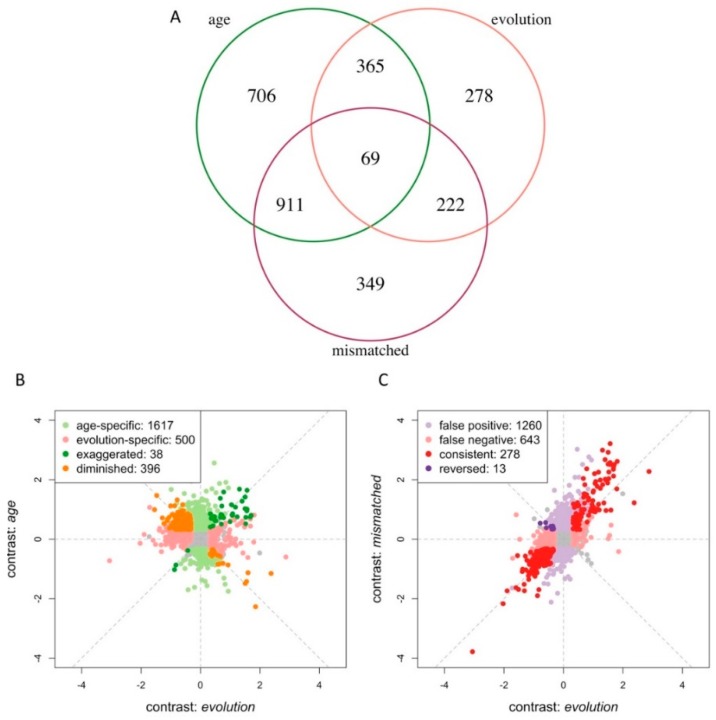
Age confounds the adaptive response in gene expression. (**A**) Three-way Venn diagram of genes identified by different contrasts. (**B**) Scatter plot of expression changes ($\log_{2}FC$) in the contrast *evolution* (*x*-axis) and the contrast *age* (*y*-axis). Although some adaptive genes were not affected by age (light red), as many as 434 adaptive genes (46%) were affected by age difference. While the age difference may have exaggerated the true signals of adaptive response (dark green), it mostly masked true adaptive signals (dark orange). In addition, 1617 genes showed significant age difference-specific expression changes (light green). (**C**) Scatter plot of expression changes ($\log_{2}FC$) in the contrasts *evolution* (*x*-axis) and *mismatched* (*y*-axis). Consistent true signals are shown with dark red dots, while false positive and false negative signals are indicated in light purple and red, respectively. In a small set of genes, the expression was even significantly reversed between the two contrasts (dark purple).

We compared the expression changes of the contrasts *age* and *evolution*. Of particular interest are 434 genes exhibiting differential expression in both contrasts (Figure 3A). Depending on the direction of change, unaccounted age-related gene expression change resulted in genes with exaggerated (38 genes) or diminished (396 genes) expression differences in the *evolution* contrast (Figure 3B). As a consequence, it is possible that either evolved differences in gene expression were masked by age difference or, in some extreme cases, the age difference resulted in false adaptive expression changes in the opposite direction. In addition, we obtained 1617 genes that were private to *age* contrast. These genes could result in putative false positives (Figure 3A).

**Figure 4:**
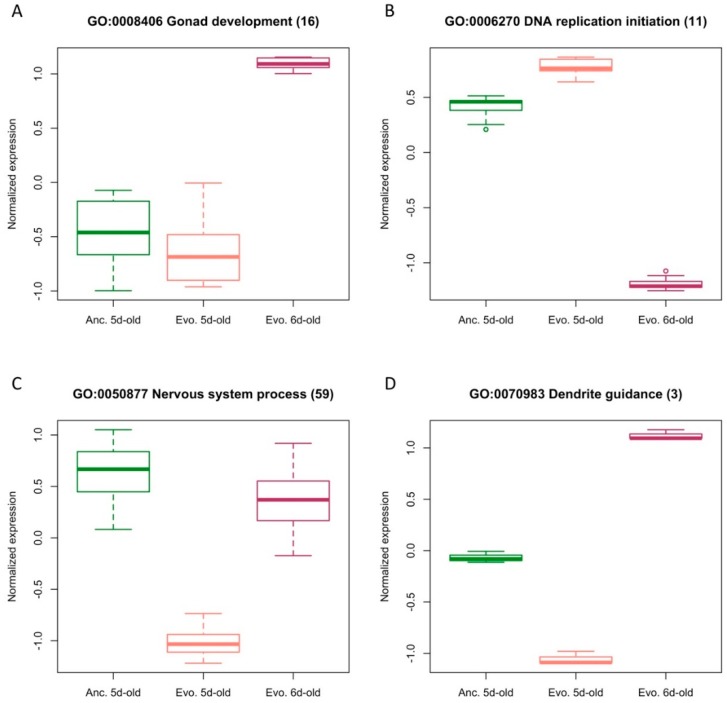
Expression changes for inconsistent genes in the contrasts *mismatched* and *evolution* for different biological processes. Normalized expression of genes differentially expressed in the contrast between evolved and ancestral flies differing in age and enriched in different GO categories. (**A**) False upregulation; (**B**) false downregulation; (**C**) false negative signatures; (**D**) reversed expression pattern.

To explicitly evaluate how age affects the inference of gene expression evolution in response to temperature adaptation, we investigated the expression changes in a contrast of ancestral and evolved flies differing 24 h in age (contrast: *mismatched*). In this contrast, 1551 genes were differentially expressed, and only 291 of them coincided with the contrast involving flies of the same age (Figure 3A). Additionally, 1260 genes were private to the contrast *mismatched* and are involved in a wide range of processes related to tissue development (e.g., gonad development (GO:0008406), Figure 4A) and cell cycle-related processes (e.g., DNA replication initiation (GO:0006270), Figure 4B). Most of these GO categories were also detected in the age contrast (Table S3). Out of 1260 genes private to the contrast *mismatched*, 911 (72.3%) were significant in the contrast *age* (Figure 3A and Figure S3). This shows that the age difference introduced a substantial number of false positives in the *mismatched* contrast. On the other hand, age also resulted in a large number of false negatives: around 70% (643) of the genes with significant differences in the evolution contrast were missed in the *mismatched* contrast (Figure 3A). These genes are involved in signal transduction (GO:0007165) and nervous system process (GO:0050877) (Figure 4C, Table S3). About half of these 643 false negatives can be explained by age-related gene expression differences in the opposite direction than evolution (Figure S3). Out of 291 genes identified in the *evolution* and *mismatched* contrasts, 13 genes changed expression in the opposite direction (Figure 3C). Three of these genes are involved in dendrite guidance (GO:0070983, *p* = 5.1 × 10^−6^) (Figure 4D, Table S3). 

## 4. Discussion

Contrasting evolved populations with 24 h age difference, i.e., at day five and day six after eclosure, provides two interesting insights into short-term expression changes. First, it was surprising to find such a large number of differentially expressed genes. Second, the significant overlap of GO category enrichment and differentially expressed genes between short-term and long-term aging is remarkable (Table 1). Hence, we conclude that in our experimental conditions flies display, already in 24 h, expression signatures of aging. 

**Figure 5:**
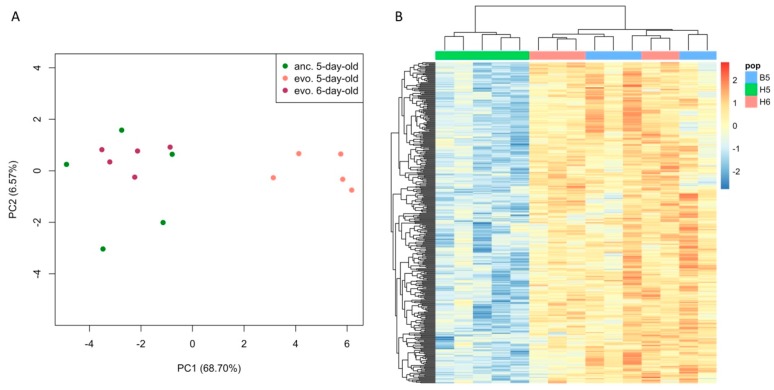
Gene expression pattern of the 374 adaptive genes with diminished signals due to 24 h age difference. (**A**) Principal component analysis (PCA) based on the expression pattern of these 374 genes. Among these genes, we found very little expression difference between 5-day-old ancestral populations and 6-day-old evolved populations, but we observed a clear distinction for 5-day-old evolved flies. (**B**) Heatmap of the normalized expression pattern of all 374 genes in this category. Lower expression was seen in 5-day-old evolved samples (H5), while similar high expression was observed in 5-day-old ancestors (B5) and 6-day-old evolved flies (H6).

In addition, 434 of the genes with significant gene expression changes were shared in the *age* and *evolution* contrasts. Almost half of all genes that evolved in gene expression during 100 generations of temperature adaptation were also significantly affected by age. The majority (374, 85%) of them were downregulated during evolution but upregulated by age (Figure 3A). A PCA and cluster analysis of these genes clearly separated the 5-day-old evolved flies from the two other groups (Figure 5), suggesting that the expression of at least a subset of genes involved in the early aging process was delayed in flies adapting for 100 generations to the novel hot environment. These genes are enriched for several developmental processes (Table S2), including mushroom body development (GO:0016319), eye-antennal disc development (GO:0035214), and somatic muscle development (GO:0007525). Although our results suggest that temperature adaptation is mediated by or coincides with a modified aging processes, further experiments incorporating phenotypic assays at more time points in evolved and ancestral populations are needed to explicitly investigate the evolution of aging processes in the novel temperature regime.

Our study showed that already a small difference in age of only 24 h results in significant gene expression differences. This observation not only has important implications for the design of future gene expression profiling studies but also triggers re-consideration of some published results as the mixture of flies with different age corresponds to the combination of different expression patterns (Figure 4). If such mixtures are not well-balanced among the focal groups, this can result in false positives and negatives. Unless the distribution of age classes and their specific gene expression patterns are known, it will not be possible to correct for these mixture effects. Hence, we recommend that future studies should minimize age differences between pooled specimen. Alternatively, expression profiling of individuals with documented age may provide an analytical framework, which permits the incorporation of age as an additional factor.

## Figures and Tables

**Table 1:** Consistency of gene expression changes in short- (24 h) and long- (up to 85 days) term aging.

	Short-Term ^1^	Long-Term ^2^ [33]	Consistency ^3^
Genes	2051	966	366 (37.9%) ***
GO terms	312	89	24 (27.0%) ***

^1^ Numbers of age-related genes and enriched gene ontology (GO) terms in this study. ^2^ Numbers of age-related genes with corresponding ID and enriched GO terms in reference [33]. ^3^ Numbers of genes and GO terms consistently identified in both short- and long-term aging *** Fisher’s exact test, *p* < 0.05.

## Supplementary Materials

The material is available online at http://www.mdpi.com/2073-4425/10/2/89/s1, Figure S1: Venn diagram of genes identified in different contrasts by DESeq2 and edgeR, Figure S2: Magnitude of expression changes by age difference and evolution, Figure S3: Pie chart showing the proportion of age-related effect among the differentially expressed genes in the contrast *mismatched*. Table S1: mapping statistics, Table S2: GO enrichment analysis of contrasts between ancestral and evolved samples of same age and between genetically similar samples of 24 h age difference, Table S3: GO enrichment analysis of contrasts between ancestral and evolved samples of mismatched age.
