# Supplementary material for: A 24 h Age Difference Causes Twice as Much Gene Expression Divergence as 100 Generations of Adaptation to a Novel Environment
Source: Genes (Basel). 2019 Jan 28;10(2):89. doi: 10.3390/genes10020089 (PMC6410183; doi:10.3390/genes10020089)
Supplement: Supplementary file 1 [file genes-10-00089-s001.zip › genes-429923-SI/genes-429923 supp figures.pdf]

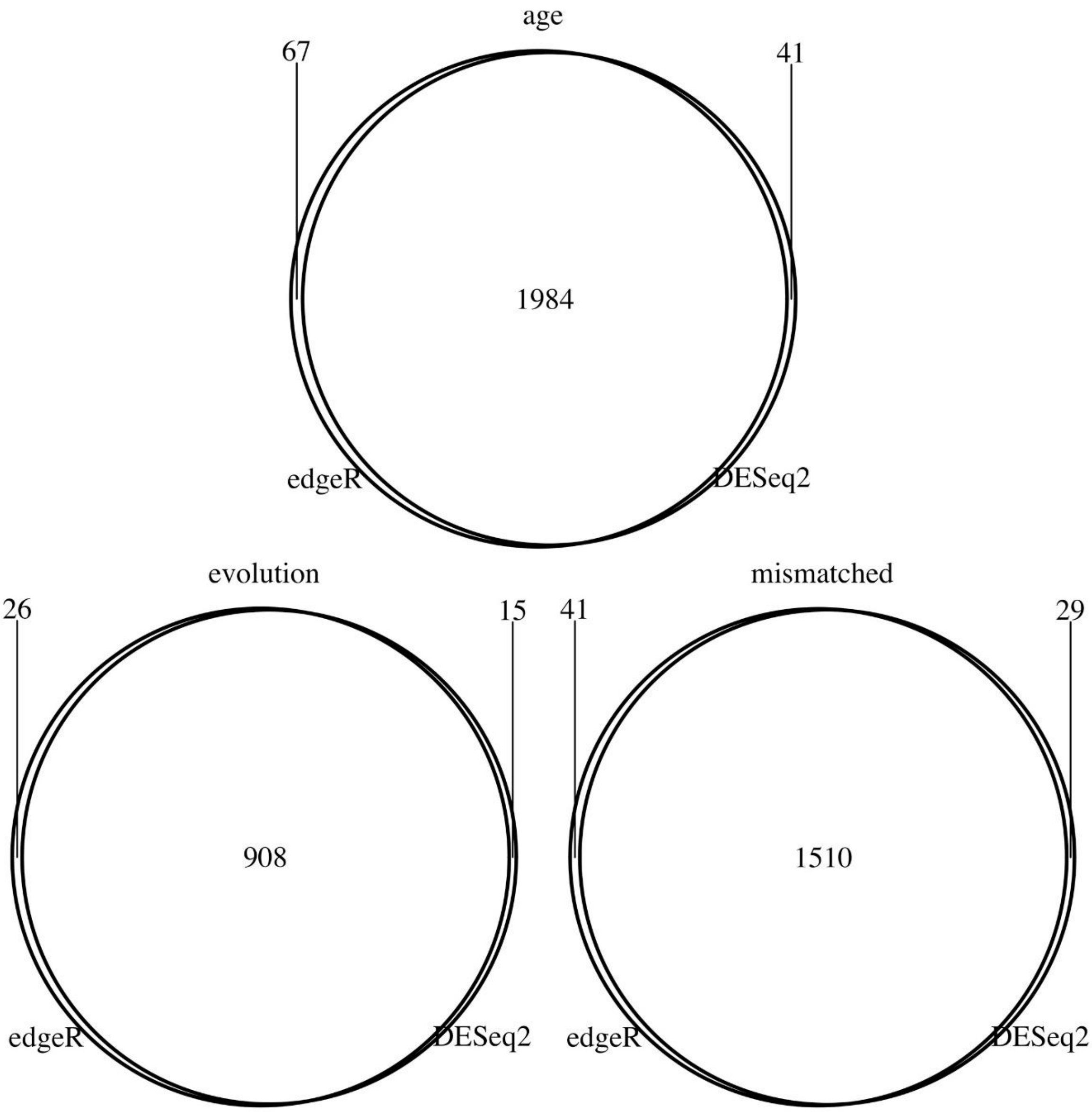

Figure S1: Venn diagram of genes identified in different contrasts by DESeq2 and edgeR.

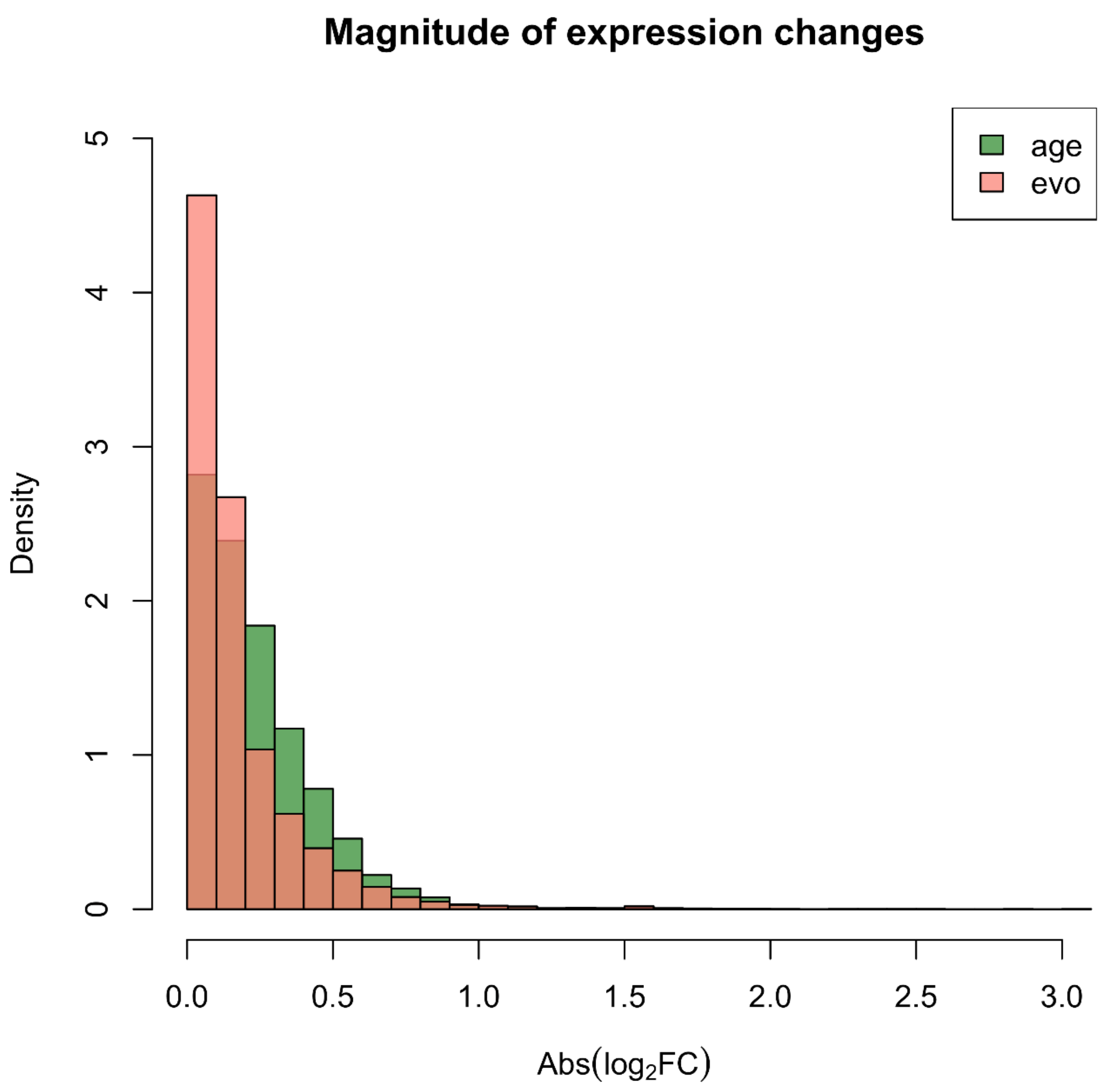

Figure S2: Magnitude of expression changes by age difference and evolution.

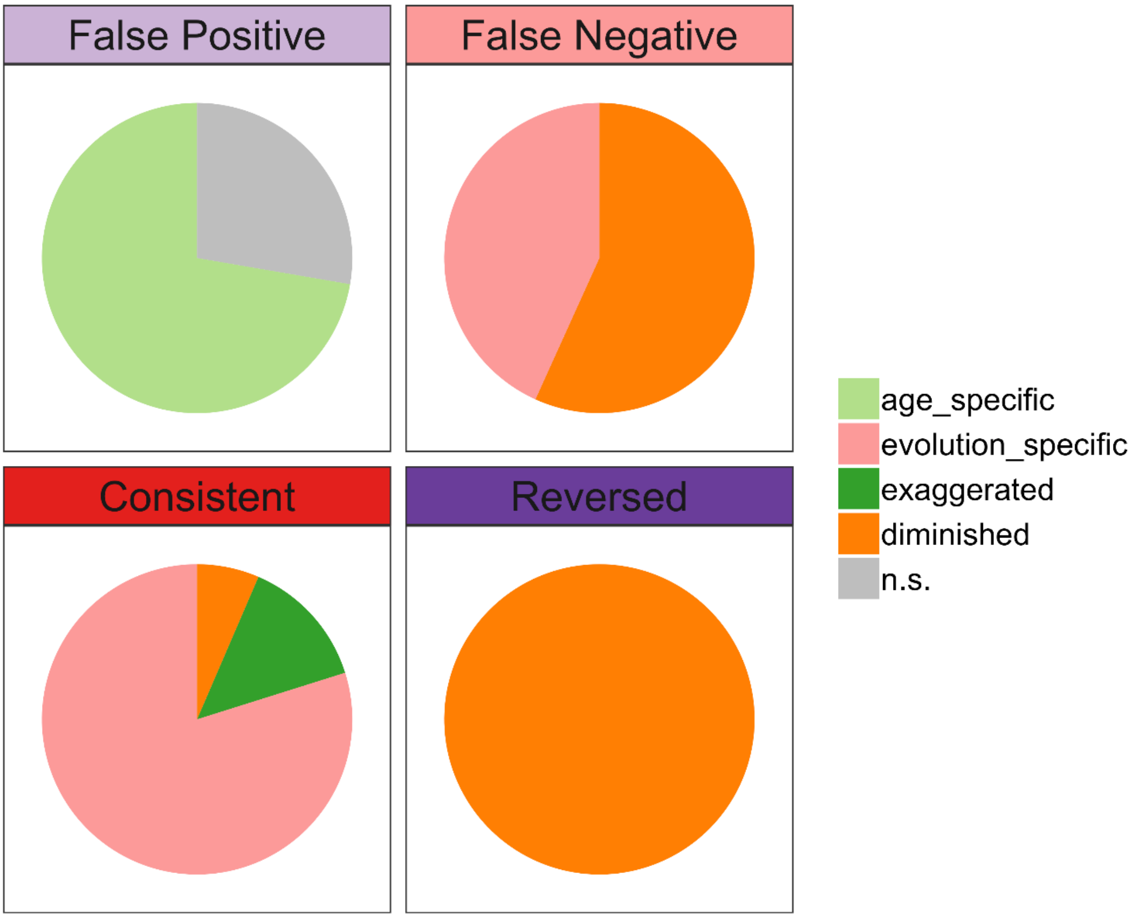

Figure S3: Pie chart showing the proportion of age-related effect among the differentially expressed genes in the contrast mismatched.
